# On the pathways feeding the H_2_ production process in nutrient-replete, hypoxic conditions. Commentary on the article “Low oxygen levels contribute to improve photohydrogen production in mixotrophic non-stressed *Chlamydomonas cultures*”, by Jurado-Oller et al., Biotechnology for Biofuels, published September 7, 2015; 8:149

**DOI:** 10.1186/s13068-017-0800-6

**Published:** 2017-05-04

**Authors:** Alberto Scoma, Szilvia Z. Tóth

**Affiliations:** 1 Center for Microbial Ecology and Technology (CMET), Faculty of Bioengineering, University of Gent, Coupure Links 653, 9000 Ghent, Belgium; 2 Biological Research Centre Szeged, Hungarian Academy of Sciences, Temesvári krt. 62, Szeged, 6726 Hungary; 3 Center for Geomicrobiology, Aarhus University, Ny Munkegade 116, 8000 Aarhus, Denmark

**Keywords:** Acetate, Biophotolysis, *Chlamydomonas reinhardtii*, Fermentation, Hypoxia, Hydrogenase, H_2_ production, Green alga, Photosystem II, Respiration

## Abstract

**Background:**

Under low O_2_ concentration (hypoxia) and low light, *Chlamydomonas* cells can produce H_2_ gas in nutrient-replete conditions. This process is hindered by the presence of O_2_, which inactivates the [FeFe]-hydrogenase enzyme responsible for H_2_ gas production shifting algal cultures back to normal growth. The main pathways accounting for H_2_ production in hypoxia are not entirely understood, as much as culture conditions setting the optimal redox state in the chloroplast supporting long-lasting H_2_ production. The reducing power for H_2_ production can be provided by photosystem II (PSII) and photofermentative processes during which proteins are degraded via yet unknown pathways. In hetero- or mixotrophic conditions, acetate respiration was proposed to indirectly contribute to H_2_ evolution, although this pathway has not been described in detail.

**Main body:**

Recently, Jurado-Oller et al. (Biotechnol Biofuels 8: 149, 7) proposed that acetate respiration may substantially support H_2_ production in nutrient-replete hypoxic conditions. Addition of low amounts of O_2_ enhanced acetate respiration rate, particularly in the light, resulting in improved H_2_ production. The authors surmised that acetate oxidation through the glyoxylate pathway generates intermediates such as succinate and malate, which would be in turn oxidized in the chloroplast generating FADH_2_ and NADH. The latter would enter a PSII-independent pathway at the level of the plastoquinone pool, consistent with the light dependence of H_2_ production. The authors concluded that the water-splitting activity of PSII has a minor role in H_2_ evolution in nutrient-replete, mixotrophic cultures under hypoxia. However, their results with the PSII inhibitor DCMU also reveal that O_2_ or acetate additions promoted acetate respiration over the usually dominant PSII-dependent pathway. The more oxidized state experienced by these cultures in combination with the relatively short experimental time prevented acclimation to hypoxia, thus precluding the PSII-dependent pathway from contributing to H_2_ production.

**Conclusions:**

In *Chlamydomonas*, continuous H_2_ gas evolution is expected once low O_2_ partial pressure and optimal reducing conditions are set. Under nutrient-replete conditions, the electrogenic processes involved in H_2_ photoproduction may rely on various electron transport pathways. Understanding how physiological conditions select for specific metabolic routes is key to achieve economic viability of this renewable energy source.

## Background

The green microalga *Chlamydomonas reinhardtii* (hereafter referred to as *Chlamydomonas*) has been largely investigated for its capacity to generate molecular H_2_ (reviewed in [1]). Under anaerobic conditions, chloroplast-located [FeFe]-hydrogenase enzymes (HYDA1 and HYDA2) function as acceptors for electrons derived mostly from the photosynthetic electron transport. This activity prevents the over-reduction of electron carriers in the transport chain under the induction of photosynthesis and ultimately supports ATP formation. H_2_ production acts as a safety valve releasing excess reducing power by the cell, and represents a survival strategy under stressful conditions.

The discovery that the lack of nutrients such as sulfur from Tris–Acetate–Phosphate (TAP) medium enhances H_2_ production capacity in *Chlamydomonas* has laid the basis for dozens of papers which describe the elaborate interplay of metabolic routes operating under these conditions [2]. Electrons feeding the H_2_ production process may derive from the PSII water-splitting activity, from mobilization of internal reserves entering the electron transport chain at the level of the plastoquinone (PQ) pool (e.g., starch) or through dark fermentative pathways (reviewed in [1]). Despite great advances in the field, this biotechnology is still far from being applicable. The time-consuming and expensive sulfur-deprivation step and its dependence on acetate limit its feasibility to laboratory conditions, with only three studies in the literature that used solar light and/or outdoor photobioreactors (PBRs) (reviewed in [3]).

Another major constraint in the application of nutrient-deprived media is that algal metabolism is highly impacted, leading to cell lysis and death unless the cell culture is re-supplemented with nutrients.

H_2_ production can also be induced under nutrient-replete conditions. Upon illumination of dark-adapted cultures, H_2_ production is normally limited to few minutes, as the O_2_ evolved by PSII in the light ends up irreversibly inhibiting [FeFe]-hydrogenases. Dark anaerobic incubation, i.e., flushing the culture with nitrogen or argon gas for a few hours in darkness, promotes the expression of hydrogenases, and in moderate light H_2_ production is extended to 1–2 h [4]. Attempts at further prolonging incubation in the light resulted in the establishment of hypoxic conditions, with H_2_ production lasting for days [5, 6], although at moderate efficiencies as compared to nutrient-deprived cultures [6]. While much information has been obtained on the electron transport processes operating in nutrient-deprived conditions, much less is known about hypoxia and the pathways supporting long-term H_2_ production under nutrient-replete conditions.

The present Commentary discusses the recent publication by Jurado-Oller et al. [7] describing the process of H_2_ production attained at low O_2_ concentrations in complete TAP medium, with a focus on the potential electron sources and physiological conditions allowing H_2_ production.

## Main text

### Acclimation to hypoxia in nutrient-replete conditions and excess reducing power

The first study to investigate the effects of prolonged hypoxia in the light in *Chlamydomonas* cultures was reported by Degrenne et al. [5] using the wild-type strain 137AH. Establishment of a specific light-illuminated fraction within the PBR notably reduced the O_2_ concentration in the culture and allowed H_2_ evolution. After acclimation to hypoxia (between 4 and 15 days, depending on the experimental set-up, see Figs. 2B, 3B and 4B in [5]), a burst in H_2_ production was observed (with light intensities between 50 and 135 µmol photons m^−2^ s^−1^). The 3- to 4-day long H_2_ production was accompanied by a reduction in protein content and a strong decrease of PSII photochemical efficiency (F_v_/F_m_) (Figs. 3C and 4B in [5]). The latter value was restored to about 0.3 as H_2_ production ceased, along with slowly increasing O_2_ concentrations, indicating that photosynthesis was partially recovered (Fig. 3C in [5]). The amount of starch did not change over the course of the experiment and its contribution to the process was considered null.

Later on, Scoma et al. [6] tested acclimation to hypoxia under photoheterotrophic conditions using the D1 protein mutant strain L159I-N230Y. Here, low O_2_ concentrations were set in the PBR by balancing the photosynthesis: respiration ratio, that is, by incubating dense cultures (between 40 and 80 mg chlorophyll a + b [Chl] L^−1^) at low light intensity (20 µmol photons m^−2^ s^−1^). These conditions are light-limiting for algal cells. Following an initial burst of H_2_ production in the first 24 h, acclimation to hypoxia took place in 10 days, after which a constant H_2_ accumulation occurred over a period of more than 14 days (Figs. 2 and 4 in [6]). Reducing power contributing to H_2_ production was proposed to arise from PSII activity (biophotolysis) and proteins through photofermentation, with an indirect contribution from acetate. The amount of starch per cell increased over the course of the H_2_ production phase, indicating that both biomass and H_2_ could potentially be produced at the same time (Fig. 3 in [6]).

Notwithstanding the different set-up, these two investigations showed some common features for the H_2_ production achieved under hypoxic conditions in nutrient-replete media: (1) protein degradation may contribute to the H_2_ production process via yet unknown pathways, (2) reduction in PSII photochemical efficiency coincided with the start of sustained H_2_ gas production both in the absence and presence of acetate, supplied as an organic carbon source (autotrophy in Degrenne et al. [5], and mixotrophy in Scoma et al. [6]). While the transient decrease of F_v_/F_m_ in Degrenne et al. [5] coincided with the 3- to 4-day long H_2_ release, in Scoma et al. [6] the effective photochemical efficiency of PSII in the light (ΔF/F_m_′) followed an antiparallel pattern with respect to H_2_ accumulation.

These observations support the hypothesis formulated by Scoma et al. [6] that a balanced photosynthesis: respiration ratio is a prerequisite for the establishment of hypoxia, resulting in a reduced PQ-pool and thus a low photochemical operating efficiency. The reason behind this is that electrons are continuously fed to the PQ-pool via respiration, and the plastid terminal oxidase (PTOX) responsible for PQ-pool oxidation is O_2_-dependent; moreover, at low light intensities PSI is also inefficient in oxidizing the PQ-pool. The physiological role of H_2_ production in the cell is to act as a safety valve; thus, the over-reduced photosynthetic electron transport chain stimulates the expression of the hydrogenase enzyme (Table 2; Fig. 6 in [6]), via a high proton gradient around PSI and a reduced PQ-pool ([8] and references therein). While continuous H_2_ production through a PSII-dependent pathway is accompanied by low PSII photochemical operating efficiency [6], transient H_2_ productions are followed by a (partially) restored PSII efficiency [5], possibly due to the transient re-oxidation of the photosynthetic electron transport chain. This mechanism resembles the findings of Antal et al. [9] under sulfur deprivation, with increasing ΔF/F_m_′ values coinciding with the onset of H_2_ production.

### The contribution of acetate respiration to H_2_ production under nutrient-replete conditions

Jurado-Oller et al. [7] investigated the effect of acetate in TAP medium on the H_2_ production of the *Chlamydomonas* strain 704 (cw15 arg7^+^ Nia1:Ars mt^+^). Different O_2_ levels were set and their impact on acetate consumption, starch accumulation, and H_2_ output were compared. In normal, non-aerated cultures kept at low light intensities (12–50 µmol photons m^−2^ s^−1^), the cells produced H_2_ mostly in the first 24 h, followed by H_2_ consumption during the 10-day experiment; at 100 µmol photons m^−2^ s^−1^ no H_2_ was evolved.

In the earlier report by Scoma et al. [6], adaptation to hypoxia took place in the first 10 days, during which *Chlamydomonas* cells underwent critical physiological changes (Fig. 1 and Table 1 in [6]) that allowed a long-lasting H_2_ production. The culture conditions differed slightly between Jurado-Oller et al. and Scoma et al., as the initial Chl content was 10 mg L^−1^ in Jurado-Oller et al. (Fig. S1 in [7]), i.e., four times less than the initial Chl content in Scoma et al. [6], whereas the light intensity was higher (12–100 µmol photons m^−2^ s^−1^ vs. 20 µmol photons m^−2^ s^−1^ in Scoma et al. [6]). Both light intensity and Chl (or biomass) content affect the establishment of hypoxia in the culture, thereby impacting H_2_ production pathways. The high biomass content and low light used in Scoma et al. served three purposes: (1), to reduce the O_2_ output; (2), to increase the respiration capacity per volume of PBR thus preventing O_2_ accumulation, which results in a more reduced state of the PQ-pool facilitating hydrogenase expression and H_2_ production; (3) to slow down growth, thereby channeling the reducing power generated by the photosynthetic electron transport to H_2_ production. The co-occurrence of these processes allowed adaptation to hypoxia, after which a sustained 14-day long H_2_ production was observed that mostly relied on PSII activity [6].

A dependence of H_2_ production on acetate respiration was suggested by Jurado-Oller et al. [7] and potential pathways accounting for the acetate contribution to H_2_ production were suggested (Fig. 6 in [7]). The authors surmised that acetate would enter the glyoxylate pathway, generating succinate as metabolic intermediate. The latter would be oxidized by a succinate dehydrogenase (SDH) in the chloroplast, reducing FAD^2+^ to FADH_2_, which in turn may reduce the PQ-pool and enter the electron transport chain. Alternatively, malate also resulting from the glyoxylate pathway could be oxidized to oxaloacetate by a malate dehydrogenase (MDH) located in the chloroplast, thereby reducing NADP^+^ to NADPH, with the latter oxidized by the PQ-pool and entering the H_2_ production pathway. Finally, oxaloacetate itself may be oxidized in the dark by a pyruvate:ferredoxin reductase (PFR), entering the dark fermentative pathway as proposed in [10].

The conclusion that acetate respiration contributes significantly to H_2_ production was based essentially on two types of experiments by Jurado-Oller et al. [7]. When the cultures were “aerated” (i.e., some O_2_ was added by opening the vials), the amount of H_2_ produced and the rate of acetate consumption increased, which were both essentially DCMU-insensitive (only 10–30% decrease upon DCMU treatment as compared to low light, Fig. 2A and E in [7]). The other experiment consisted of adding 8.5 mM acetate to the cultures every 4 days, which resulted in a DCMU-insensitive increase in H_2_ production (Fig. 4A and E in [7]). Based on these results, the authors conclude that “H_2_ production in LL (low light) mixotrophic cultures is mainly via PSII-independent pathways.” The acetate respiration supporting H_2_ production may have operated through SDH and MDH, thus producing FADH_2_ and NADH. This pathway would be independent of PSII activity, as it only requires photosystem I (PSI) to transfer electrons to the hydrogenase.

Notwithstanding the potential indirect contribution from acetate respiration, this conclusion contradicts their observations that the addition of DCMU to normal non-aerated cultures irradiated with 12 µmol photons m^−2^ s^−1^ resulted in a 72.4% decrease in H_2_ production (compare Fig. 1A and E in [7]). While the authors agree that “the level of inhibition of H_2_ production by DCMU is subjected to the specific culture conditions”, they do not recognize that their operating conditions were unfavorable for a PSII-based H_2_ production. In cultures with relatively low biomass, exposed to O_2_ and high acetate levels, respiration pathways are very likely to be stimulated. These conditions may have lowered the activity of the PSII-dependent pathway. Provided that O_2_ was available, acetate respiration and its potential contribution to H_2_ production were enhanced. Unfortunately, the photochemical efficiency of PSII (either F_v_/F_m_ or ΔF/F_m_′) was not measured, neither was the in vitro hydrogenase activity. Differences in cellular redox potentials may also explain why cultures tested under identical light regimes achieved 1.9 and 3.2% H_2_ partial pressure in the presence and absence of elemental sulfur, respectively (compare low light in Fig. 1A and Fig. S3A in [7]). In addition, some cultures consumed H_2_ gas although its concentration was below 5% H_2_ partial pressure, which has been shown to be the critical threshold between H_2_ production and oxidation [11].

Significant metabolic changes may occur once PSII-dependent electron transport is blocked in hypoxia-acclimated cells as by DCMU addition. In Scoma et al. [6], these cells produced H_2_ in the light and in the dark (Fig. 4 and Table 2 in [6]) while consuming acetate and producing a minor amount of formate and ethanol (Fig. 5 in [6]). DCMU treatment of hypoxia-acclimated cells resulted in no additional acetate consumption, but enhanced succinate, formate, and ethanol production, which were independent on illumination (Fig. 5 in [6]). These results demonstrate that the contributions of the various metabolic pathways to H_2_ production may largely vary depending on the culture conditions. Enhanced respiration rates upon O_2_ and acetate addition in cells tested in hypoxia by Jurado-Oller et al. [7] may have altered these fermentative pathways, with DCMU treatment further impacting the intermediate metabolites possibly entering the H_2_ production pathway.

## Conclusions

H_2_ metabolism operating in hypoxic conditions in *Chlamydomonas* does not appear to be less complicated than the one activated upon sulfur deprivation. Depending on the conditions, competition for reduced ferredoxin in nutrient-replete conditions may favor several electron acceptors other than hydrogenase, driving much reducing power out of the H_2_ production pathway. Culture conditions also have an impact on the metabolic routes supporting H_2_ production. Since H_2_ production is primarily a safety valve releasing excess reducing power, analysis of the cellular redox state may provide critical information on the contribution of the different pathways supporting the process. Addition of specific inhibitors and nutrients may enhance the contribution of pathways that previously had a minor role.

Based on the recent data by Jurado-Oller et al., it can be concluded that during H_2_ production in nutrient-replete conditions:

1. Electrons derived from acetate respiration which are not used for growth may enter the H_2_ production process;
2. The presence of O_2_ may result in an increased contribution of acetate respiration to H_2_ production.

Jurado-Oller et al. [7] proposed that cell cultures in hypoxia may potentially use two possible pathways for acetate respiration in the light, which involve succinate and/or malate. Metabolomics and/or analysis of the transcriptomic or proteomic response under different culture conditions may clarify which one is responsible for H_2_ production. Integration of such data with hydrogenase expression and PSII photochemical efficiency should also highlight which physiological condition triggers one pathway or another under nutrient-replete, hypoxic conditions. In particular, the following questions remain unanswered regarding acetate respiration supporting H_2_ evolution:

1. What is the photochemical efficiency of PSII and the in vitro hydrogenase expression level and activity when acetate respiration contributes substantially to H_2_ evolution?
2. Which metabolites are actually produced (if any) during H_2_ evolution in the light and in the dark?
3. What is the acetate-to-H_2_ energy conversion efficiency as compared to acetate-to-biomass?
4. What are the alternative electron sinks at the PSI acceptor side?

Based on the available data, the present protocol by Jurado-Oller et al. [7] may represent less stressful conditions as compared to the ones described in Degrenne et al. [5] or Scoma et al. [6], and has the potential to be operated for a longer period of time, although H_2_ production yields remain still very low relative to cultures deprived of sulfur. However, the usage of photosynthetic microalgae through a PSII-independent pathway may not be a viable option for H_2_ production as compared to microbial fermentative biotechnologies using bacterial consortia and biowastes. Therefore, research efforts with oxygenic photosynthetic microbes should be directed at promoting PSII-dependent pathways using water as an electron source.

## Abbreviations

Chl: chlorophyll *a* + *b*
DCMU: (3-(3,4-dichlorophenyl)-1,1-dimethylurea)
ΔF/F_m_’: effective photochemical efficiency of PSII
F_v_/F_m_: maximum photochemical efficiency of PSII
MDH: malate dehydrogenase
PBR: photobioreactor
PFR: pyruvate:ferredoxin reductase
PQ-pool: plastoquinone pool
PSI: photosystem 1
PSII: photosystem 2
SDH: succinate dehydrogenase
TAP: Tris–Acetate–Phosphate
